# *Panax Notoginseng* flower saponins (PNFS) inhibit LPS-stimulated NO overproduction and iNOS gene overexpression *via* the suppression of TLR4-mediated MAPK/NF-kappa B signaling pathways in RAW264.7 macrophages

**DOI:** 10.1186/s13020-015-0045-x

**Published:** 2015-07-01

**Authors:** Xiao-Xu Peng, Shu-Hui Zhang, Xiao-Ling Wang, Ting-Jie Ye, Hua Li, Xiao-Feng Yan, Li Wei, Zhong-Ping Wu, Jing Hu, Chun-Pu Zou, You-Hua Wang, Xu-Dong Hu

**Affiliations:** Department of Biology, School of Basic Medical Science, Shanghai University of Traditional Chinese Medicine, Shanghai, 201203 People’s Republic of China; Hypertension Laboratory, Cardiovascular Department, Long Hua Hospital affiliated to Shanghai University of Traditional Chinese Medicine, Shanghai, 200032 People’s Republic of China; Yueyang Hospital, Shanghai University of Traditional Chinese Medicine, Shanghai, 200437 People’s Republic of China; School of Pharmacy, Shanghai University of Traditional Chinese Medicine, Shanghai, 201203 People’s Republic of China; School of Basic Medical Science, Shanghai University of Traditional Chinese Medicine, Shanghai, 201203 People’s Republic of China

## Abstract

**Background:**

*Panax Notoginseng* flower saponins (PNFS) are the main active component of *Panax notoginseng* (Burk) F. H. Chen flower bud (PNF) and possess significant anti-inflammatory efficacy. This study aims to explore the mechanisms underlying PNFS’ antiflammatory action in RAW264.7 macrophages.

**Methods:**

A cell counting kit-8 assay was used to determine the viability of RAW264.7 macrophages. Anti-inflammation effects of PNFS in lipopolysaccharide (LPS)-stimulated RAW264.7 macrophages were measured based on the detection of nitric oxide (NO) overproduction (Griess method, DAF-FM DA fluorescence assay and NO_2_^−^ scavenging assay), and interleukin (IL)-6 and tumor necrosis factor (TNF)-alpha gene overexpression (real-time PCR and ELISA). Inducible nitric oxide synthase (iNOS) gene overexpression was determined by real-time PCR and western blotting. iNOS enzyme activity was also assayed. The mechanisms underlying the suppression of iNOS gene overexpression by PNFS were explored using real-time PCR and western blotting to assess mRNA and protein levels of components of the Toll-like receptor 4 mitogen-activated protein kinase (MAPK), phosphatidylinositol 3-kinase (PI3K)/Akt, and nuclear factor-kappa B (NF-kappa B) signaling pathways.

**Results:**

PNFS (50, 100, 200 μg/mL) significantly reduced LPS-induced overproduction of NO (*P <* 0.001, *P <* 0.001, *P <* 0.001) and IL-6 (*P =* 0.103, *P <* 0.001, *P <* 0.001), but did not affect TNF-alpha overproduction. PNFS (50, 100, 200 μg/mL) also markedly decreased LPS-activated iNOS (*P <* 0.001, *P <* 0.001, *P <* 0.001) and TLR4 gene overexpression (*P =* 0.858, *P =* 0.046, *P =* 0.005). Furthermore, treatment with PNFS (200 μg/mL) suppressed the phosphorylation of MAPKs including P38 (*P =* 0.001), c-Jun N-terminal kinase (JNK) (*P =* 0.036) and extracellular-signal regulated kinase (ERK) 1/2 (*P =* 0.021). PNFS (200 μg/mL) inhibited the activation of the NF-kappa B signaling pathway by preventing the phosphorylation of inhibitor of NF-kappa B alpha (I-kappa B alpha) (*P =* 0.004) and P65 (*P =* 0.023), but PNFS (200 μg/mL) could not activate the LPS-induced PI3K-Akt signaling pathway.

**Conclusions:**

PNFS significantly down-regulated iNOS gene overexpression and thereby decreased NO overproduction via the inhibition of TLR4-mediated MAPK/NF-kappa B signaling pathways, but not the PI3K/Akt signaling pathway.

## Background

*Panax notoginseng* (Burk) F. H. Chen flower bud (PNF) can be used to treat hypertension closely related to inflammatory response [1, 2], chemotherapy stomatitis, pharyngitis and other inflammatory diseases [3–5]. The methanol extract of PNF was shown to block the NF-kappa B signaling pathway and alleviate the lipopolysaccharide (LPS)-induced inflammatory response in murine macrophages [6].

*Panax notoginseng* flower saponins (PNFS), extracted from PNF, were reported to be the main bioactive constituent underlying PNF’s therapeutic effect [7]. Additionally, the flower was shown to contain most of the total saponin amount, more than is present in the root [7]. The composition of PNFS is different from that of *Panax notoginseng* saponins (PNS), extracted from the *Panax notoginseng* (Burk) F. H. Chen (PN) root [7]. PNFS lowered blood pressure in spontaneous hypertensive rats [8, 9]. Additionally, PNFS could relieve the inflammatory response *via* diminishing swelling and decreasing prostaglandin production in carrageenan-induced rat paw swelling and croton oil-induced mouse auricle inflammation, induced by proinflammatory agents [10, 11]. Therefore, the antihypertensive effect of PNFS might be partially associated with its anti-inflammatory effect.

Excessive inflammation causes the body to be overexposed to inflammatory mediators, *e.g.*, nitric oxide (NO), tumor necrosis factor (TNF)-alpha and interleukin (IL)-6, leading to cell necrosis, tissue injury and degeneration, and consequently, aggravating inflammation. Lipopolysaccharide (LPS) is a highly proinflammatory endotoxin from the outer envelope of gram-negative bacteria. Monocyte-derived macrophages, when stimulated with LPS, produce excessive inflammatory mediators such as NO, TNF-alpha and IL-6, in inflammatory response [12–14].

NO significantly influences the regulation of neurotransmission and inflammatory responses [15, 16]. In mammals, NO is generated by three different nitric oxide synthases (NOSs), namely, endothelial NOS (eNOS), neuronal NOS (nNOS), and inducible NOS (iNOS) [17]. iNOS, primarily identified in macrophages, is usually not expressed in normal conditions, but is expressed when induced by agents such as LPS and some cytokines [17]. In macrophages stimulated with LPS, iNOS produces large amounts of NO and exerts anti-inflammatory effects on the organism by killing undesired microbes and parasites [18]. However, when released at the wrong site or produced excessively *in vivo*, NO may aggravate inflammation via oxidative damage to healthy cells and tissues [19, 20]. Therefore, the suppression of iNOS gene overexpression to reduce NO overproduction is an important target of anti-inflammatory drugs.

Toll-like receptor 4 (TLR4) is an essential cell surface protein on macrophages for LPS recognition [21]. The interaction between TLR4 and LPS activates two main intracellular signaling pathways: the mitogen-activated protein kinase (MAPK)/nuclear factor-kappa B (NF-kappa B) signaling pathway and the phosphatidylinositol 3-kinase (PI3K)/Akt signaling pathway. Both are involved in iNOS gene expression [13, 22, 23].

This study aims to explore the suppressive effects of PNFS on proinflammatory mediator overexpression in LPS-activated RAW264.7 macrophages. Therefore, the mechanisms by which PNFS inhibits iNOS gene overexpression were studied through analysis of the levels of components of the TLR4, MAPK, PI3K/Akt, and NF-kappa B signaling pathways.

## Methods

### Reagents

LPS (*Escherichia coli* O55:B5) and sulfanilic acid were bought from Sigma Chemical Co., Ltd. (St.Louis, MO, USA). Dulbecco’s modified Eagle medium (DMEM) and fetal bovine serum (FBS) were purchased from Gibco BRL Co., Ltd. (Grand Island, NY, USA). Cell Counting Kit-8 (CCK-8) was purchased from Dojindo (Kumamoto, Japan) and TRIzol was purchased from Invitrogen (Carlsbad, CA, USA). RevertAid™ First Strand cDNA Synthesis Kit was purchased from Thermo Scientific (Waltham, MA, USA). SYBR ®Premix Ex Taq™ (Perfect Real Time) was purchased from Takara Biotechnology (Dalian) Co., Ltd (Dalian, China). Ethanol, N-(1-naphthyl) ethylenediamine dihydrochloride, H_3_PO_4_, sulfanilamide, NaNO_2_ and hydrochloric acid were purchased from Sinopharm Chemical Reagent Co., Ltd. (Shanghai, China). 3-Amino, 4-aminomethyl-2′, 7′-difluorescein, diacetate (DAF-FM DA) and cycloheximide (CHX) were purchased from Beyotime (Shanghai, China). Enzyme-linked immunosorbent assay (ELISA) kit for TNF-alpha was obtained from R&D Systems (Minneapolis, MN, USA) and ELISA kit for IL-6 was purchased from BD PharMingen (San Diego, CA, USA). Antibodies for GAPDH (#2118), iNOS (#2982), P38 (#8690), P-P38 (#4511), ERK (#4695), P-ERK (#4370), JNK (#9258), P-JNK (#4668), I-kappa B alpha (#4812), P-I-kappa B alpha (#2859), P65 (#4764), P-P65 (#3033), P-Akt (#4056), PI3K (#4255), anti-rabbit IgG, and HRP-linked antibody (#7074) were purchased from Cell Signaling Technology (Boston, MA, USA).

### PNFS extract

PNF was collected from Wenshan, Yunnan Province, China, and identified by Dr. Xiuming Cui, Wenshan Institute of Sanqi Research. The voucher specimens were deposited in the Pharmaceutical Laboratory, College of Pharmacy, Shanghai University of Traditional Chinese Medicine. During the preparation of total saponins, we took three factors into consideration: ethanol concentration, extraction time, and duration for one extraction course. The optimum extraction process determined using an orthogonal experiment design was refluxing PNF in 14 volumes of 70 % ethanol, three times, for 2 h each time. The macroporous resin AB-8 (diameter: height = 1:10; weight: raw material = 1:1.25) was chosen to concentrate PNF extract, and the concentration of the raw material was 0.2 g/mL. The extract was washed until the Molisch reaction disappeared, and then eluted with three column volumes of 70 % ethanol. Flow rates for absorption and elution were two column volumes per hour. Total PNF saponin extract was ready after drying and quantified to be 95 % pure using a UV spectrophotometer (UV 8453, Agilent Technologies, Santa Clara, CA, USA).

### Cell culture

Murine RAW264.7 macrophages were purchased from the Shanghai Cell Bank of Chinese Academy of Sciences (China). RAW264.7 macrophages were cultured in DMEM supplemented with 10 % FBS, 100 U/mL penicillin and 100 U/mL streptomycin. Cells were grown at 37 °C in a humidified incubator with 5 % CO_2_.

### Cell viability

A CCK-8 assay was used to determine the viability of RAW264.7 macrophages. A total of 1 × 10^5^ cells per well were grown in triplicate in 96-well plates. Cells were treated without or with PNFS (0, 25, 50, 100, 200, 400, 800 μg/mL) for 24 h, and then incubated with CCK-8 (20 μL/well) for 1.5 h, after which absorbance at 450 nm was measured.

### Measurement of nitrite overproduction in cell culture supernatant

The level of nitrite (NO_2_^−^) in the cell culture supernatant, stably generated during NO reactions, was assessed to determine the amount of NO production. First, 1 × 10^5^ Raw264.7 macrophages per well were grown in triplicate in 96-well plates. Cells were treated with LPS (1 μg/mL) and PNFS at different concentrations (0, 50, 100, 200 μg/mL) for 24 h, or untreated (control). Second, Griess reagent was prepared by mixing 0.1 % N-(1-naphthyl) ethylenediamine dihydrochloride (dissolved in ddH_2_O) and 1 % sulfanilamide (dissolved in 5 % H_3_PO_4_) in equal volume. Lastly, 100 μL of culture supernatant and 100 μL of Griess reagent were blended with a micropipette and set aside for 10 min at room temperature, then the absorbance at 540 nm was measured. A NaNO_2_ standard curve was used to calculate nitrite concentration [24].

### Measurement of intracellular NO production

DAF-FM DA was used to detect intracellular NO production. First, 1 × 10^5^ Raw264.7 macrophages per well were grown in triplicate in 96-well plates. Cells were treated with LPS (1 μg/mL) and PNFS at different concentrations (0, 50, 100, 200 μg/mL) for 24 h, or untreated (control). Then, the culture supernatant was removed and cells were incubated with DAF-FM DA (5 mM, 100 μL/well) at 37 °C for 20 min. Fluorescence values (excitation 495 nm, emission 515 nm) were measured using a Multimode Microplate Reader (Synergy™2, BioTek, Winooski, Vermont, USA).

### NO_2_^−^ scavenging assay

`A 5 mL total reaction mixture, containing 3 mL of NaNO_2_ (5 μg/mL) and 2 mL of PNFS (50, 100, 200 μg/mL dissolved by ddH_2_O) or 2 mL of ddH_2_O_2_ was incubated at 37 °C for 30 min. Then, 2 mL of 0.4 % sulfanilic acid (dissolved in 20 % hydrochloric acid) was added to the reaction mixture, which was incubated for 5 min. Lastly, 17 ml of ddH_2_O and 1 ml of 0.2 % N-(1-naphthyl) ethylenediamine dihydrochloride (dissolved in ddH_2_O) were added, mixed and incubated for 15 min, and absorbance at 540 nm was measured [25].

### Measurement of IL-6 and TNF-alpha overproduction

Raw264.7 macrophages (1 × 10^5^ per well) were grown in triplicate in 96-well plates and treated with LPS (1 μg/mL) and PNFS at different concentrations (0, 50, 100, 200 μg/mL), or untreated (control), for 24 h, to detect the inhibitory effects of PNFS on pro-inflammatory cytokines produced by LPS-activated cells. Then, culture supernatants were collected and commercial ELISA kits were used to measure IL-6 and TNF-alpha concentrations.

### Measurement of iNOS enzymatic activity

A total of 1 × 10^5^ RAW264.7 macrophages per well were grown with LPS (1 μg/mL) in triplicate in 96-well plates for 24 h. Then, cells were washed and treated with 1 μg/mL cycloheximide (CHX) and PNFS at different concentrations (0, 50, 100, 200 μg/mL) for the next 24 h. Finally, the culture supernatants were harvested to detect NO_2_^−^ content [24].

### Western blotting analysis

A total of 1 × 10^6^ RAW264.7 macrophages per well were grown in 12-well plates and treated with LPS (1 μg/mL) and PNFS at different concentrations (0, 50, 100, 200 μg/ml) for 24 h, with LPS (1 μg/mL) and PNFS at different concentrations (0, 200 μg/ml) for 3 h, or untreated (control). Then, cells were harvested on ice and washed once with ice-cold PBS. Lysis buffer with phosphatase and protease inhibitors (Sangon Biotech, China) was added to lyse the cells. After incubating on ice for 30 min, cell extracts were centrifuged at 14,463 × *g* in a refrigerated centrifuge (5418R, Eppendorf, Germany) at 4 °C for 10 min to collect cell total proteins, the amount of which was quantified using a BCA protein assay kit (Biomiga, USA). SDS-PAGE (10 %) was used to separate proteins, which were electro-transferred to PVDF membranes (Millipore, USA). Membranes were blocked with 5 % (wt/vol) dried skimmed milk for 1 h, and incubated with various specific primary antibodies, namely, anti-GAPDH, anti-iNOS, anti-P38, anti-P-P38, anti-ERK, anti-P-ERK, anti-JNK, anti-P-JNK, anti-I-kappa B alpha, anti-P-I-kappa B alpha, anti-P65, anti-P-P65, anti-PI3K and anti-P-Akt, to probe corresponding target proteins. Bound antibodies were detected using peroxidase-conjugated secondary antibodies, and the amount of bound antibody was assessed by enhanced chemiluminescence (ECL). Relative levels of target proteins were obtained based on the optical density of electrophoresis bands with GAPDH serving as an internal control.

### Real-time PCR analysis

A total of 1 × 10^6^ RAW264.7 macrophages per well were grown in 12-well plates and treated with LPS (1 μg/mL) and PNFS at different concentrations (0, 50, 100, 200 μg/mL) for 24 h, or untreated (control). The Trizol method [26] was used to isolate total RNA from the cells in each well. Then, 1 μg of total RNA was reverse transcribed into cDNA, which was amplified by real-time PCR. The 2-ΔCT method was used to analyze gene expression and beta-actin mRNA served as an internal control to quantify the levels of target mRNAs relatively. The cycling conditions were as follows: hold: 95 °C for 10 s; cycling: 95 °C for 5 s, 60 °C for 30 s, 40 cycles; melt: 65–95 °C. The sequences of primers used for reverse transcription are listed below: iNOS, F: 5′-AAGTCAAATCCTACCAAAGTGA-3′, R: 5′-CCATAATACTGGTTGATGAACT-3′; beta-actin, F: 5′-CATCACTATCGGCAATGAGC-3′, R: 5′-GACAGCACTGTGTTGGCATA-3′.

TLR4, F: 5′-GGCAGGTCTACTTTGGAGTCATTGC-3′, R: 5′-ACATTCGAGGCTCCAGTGAATTCGG-3′. TNF-alpha, F: 5′-GGCAGGTCTACTTTGGAGTCATTGC-3′, R: 5′-ACATTCGAGGCTCCAGTGAATTCGG-3′. IL-6, F: 5′-TCAGAATTGCCATTGCACA-3′, R: 5′-GTCGGAGGCTTAATTACACATG-3′.

### Statistical analysis

Data are presented as means (SD). Multiple comparisons were performed using the one-Way ANOVA test followed by Student-Newman-Keuls (SNK) and least significant difference (LSD) tests. *P* values <0.05 were considered to represent significant differences between means.

## Results

### The effect of PNFS on RAW264.7 macrophage viability

**Fig. 1:**
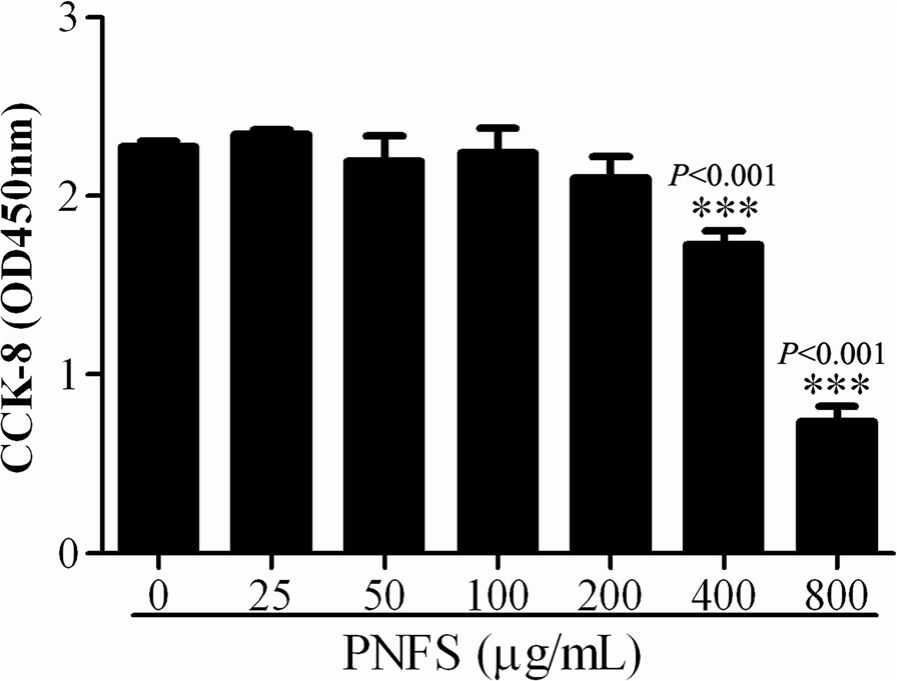
Effect of PNFS on RAW264.7 macrophage viability. Data were expressed as the mean (SD) of 3 independent experiments. One-Way ANOVA test was used to analyzed the data and the result was *F = 87.693; P < 0.001*. Then, data were counted by SNK and LSD multiple comparisons to determine the statistical difference between two groups. The *P* values represented the statistical differences between each group and negative control group (without PNFS treated). *** means *P <0.001*

To evaluate the effect of PNFS on the viability of RAW264.7 macrophages, we applied various PNFS concentrations (0–800 μg/mL) and performed a CCK-8 assay. PNFS had an obvious cytotoxic effect at 400 μg/mL (*P <* 0.001) and 800 μg/mL (*P <* 0.001), but had no cytotoxic effect at 200 μg/mL and lower concentrations on RAW264.7 macrophages (Fig. 1). So we chose the concentrations at 50, 100, 200 μg/mL for further exploring the anti-inflammatory mechanisms of PNFS.

### PNFS suppresses NO overproduction in LPS-stimulated RAW264.7 macrophages

**Fig. 2:**
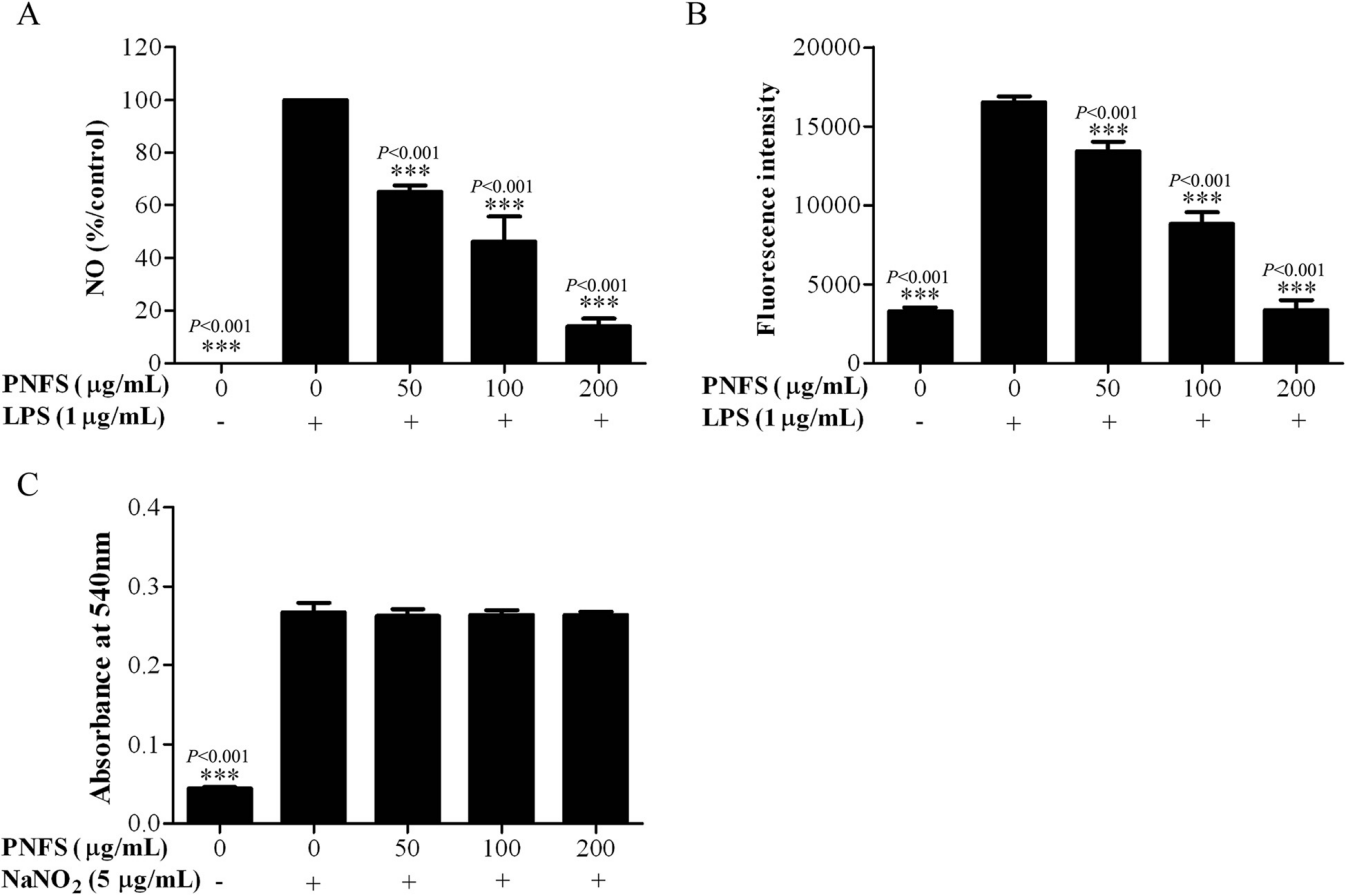
PNFS suppressed NO overproduction in LPS-stimulated RAW264.7 macrophages. **a** PNFS decreased LPS-stimulated NO overproduction in culture supernatants. **b** PNFS reduced LPS-stimulated intracellular NO level. **c** PNFS had no scavenging effect on NO_2_^−^ *in vitro*. Data in Fig. 2a, b and c were all expressed as the mean (SD) of 3 independent experiments. One-Way ANOVA test was used to analyzed the data and the results were *F = 227.437; P < 0.001*, *F = 345.932; P < 0.001* and *F = 599.919; P < 0.001*, respectively. Then, data in Fig. 2**a**, **b** and **c** were all counted by SNK and LSD multiple comparisons to determine the statistical difference between two groups. The *P* values represented the statistical differences between each group and the corresponding positive control (without PNFS and with LPS or NaNO_2_ treated). *** means *P <0.001*

The inhibitory effect of PNFS on NO overproduction in LPS-stimulated RAW264.7 macrophages was examined to evaluate the anti-inflammatory effect of PNFS. Extracellular NO concentrations were assessed by the Griess method [24] and intracellular NO concentrations were examined using a DAF-FM DA fluorescence assay. PNFS (50, 100, 200 μg/mL) markedly decreased extracellular NO concentrations (*P <* 0.001, *P <* 0.001, *P <* 0.001) and intracellular NO concentrations (*P <* 0.001, *P <* 0.001, *P <* 0.001) (Fig. 2a and b). Meanwhile, PNFS (50, 100, 200 μg/mL) did not have a NO_2_^−^ scavenging effect (Fig. 2c), which would affect the authenticity of NO detection results obtained using the Griess method. In summary, our experiments showed that PNFS suppressed LPS-stimulated NO overproduction.

### PNFS suppresses overexpression of IL-6, but exerts no influence on TNF-alpha in LPS-stimulated RAW264.7 macrophages

**Fig. 3:**
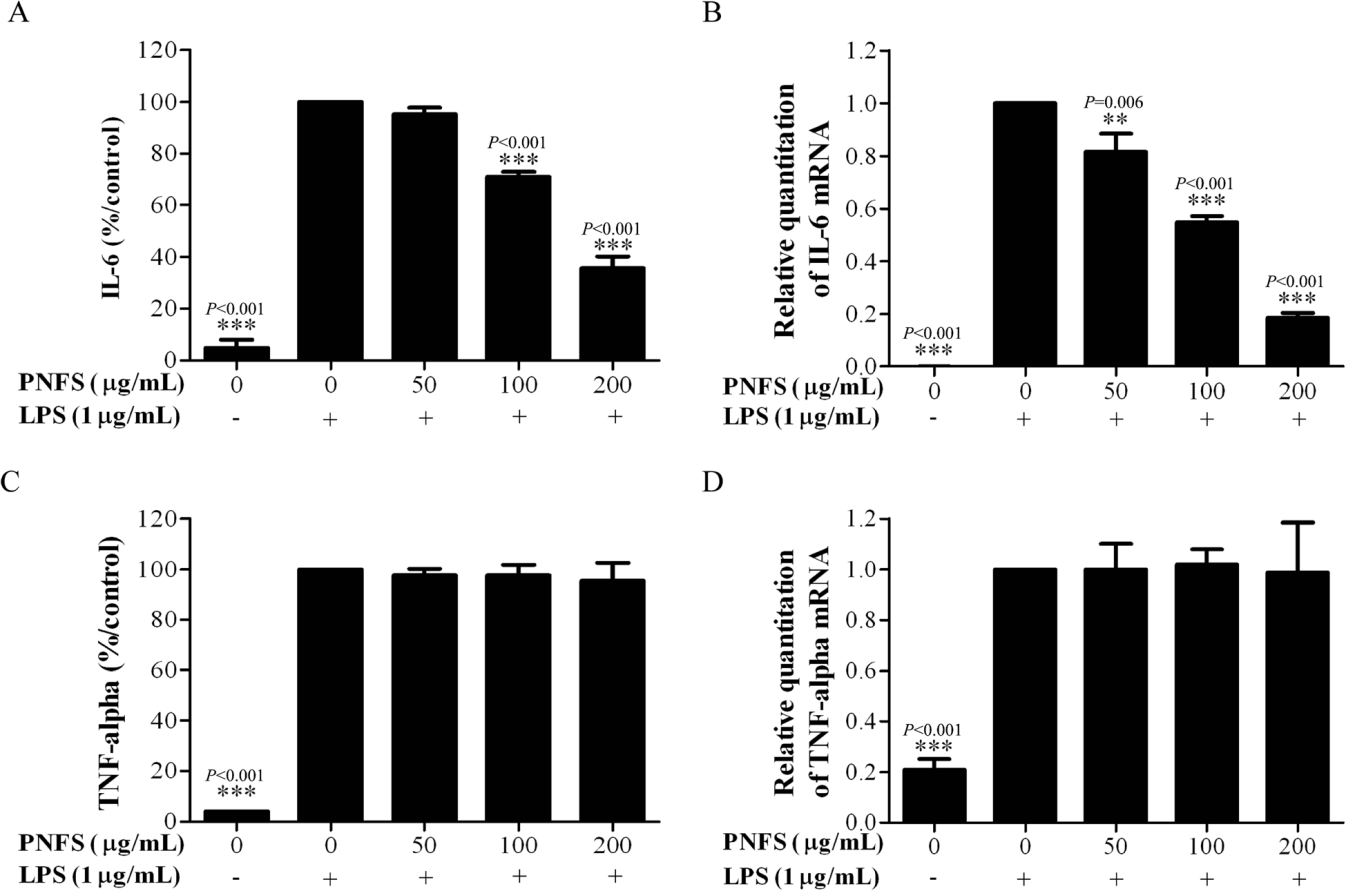
PNFS suppressed the gene overexpression of IL-6, but exerted no influence on TNF-alpha in LPS-stimulated RAW264.7 macrophages. **a**, **b** PNFS reduced LPS-stimulated IL-6 overproduction and IL-6 mRNA overexpression in RAW264.7 macrophages. **c**, **d** PNFS exerted no influence on LPS-stimulated TNF-alpha overproduction and TNF-alpha mRNA overexpression in RAW264.7 macrophages. Data in Fig. 3**a**, **b**, **c** and **d** were all expressed as the mean (SD) of 3 independent experiments. One-Way ANOVA test was used to analyzed the data and the results were *F = 766.150; P < 0.001*, *F = 124.746; P < 0.001*, *F = 212.919; P < 0.001* and *F = 7.939; P = 0.001*, respectively. Then, data in Fig. 3**a**, **b**, **c** and **d** were all counted by SNK and LSD multiple comparisons to determine the statistical difference between two groups. The *P* values represented the statistical differences between each group and the corresponding positive control (without PNFS and with LPS treated). ** means *P < 0.01* and *** means *P < 0.001*

To further explore the potential anti-inflammatory role of PNFS, we observed its effects on the overproduction of IL-6 and TNF-alpha and the overexpression of IL-6 and TNF-alpha mRNA in LPS-stimulated RAW264.7 macrophages by ELISA and real-time PCR, respectively. PNFS (50, 100, 200 μg/mL) significantly inhibited IL-6 overproduction (*P = 0.103*, *P < 0.001*, *P < 0.001*) and IL-6 mRNA overexpression (*P = 0.006*, *P < 0.001*, *P < 0.001*) (Fig. 3a and b). However, PNFS (50, 100, 200 μg/mL) exerted no influence on TNF-alpha gene overexpression (Fig. 3c and d).

### PNFS significantly inhibits iNOS gene overexpression, but does not affect iNOS enzymatic activity in LPS-stimulated RAW264.7 macrophages

**Fig. 4:**
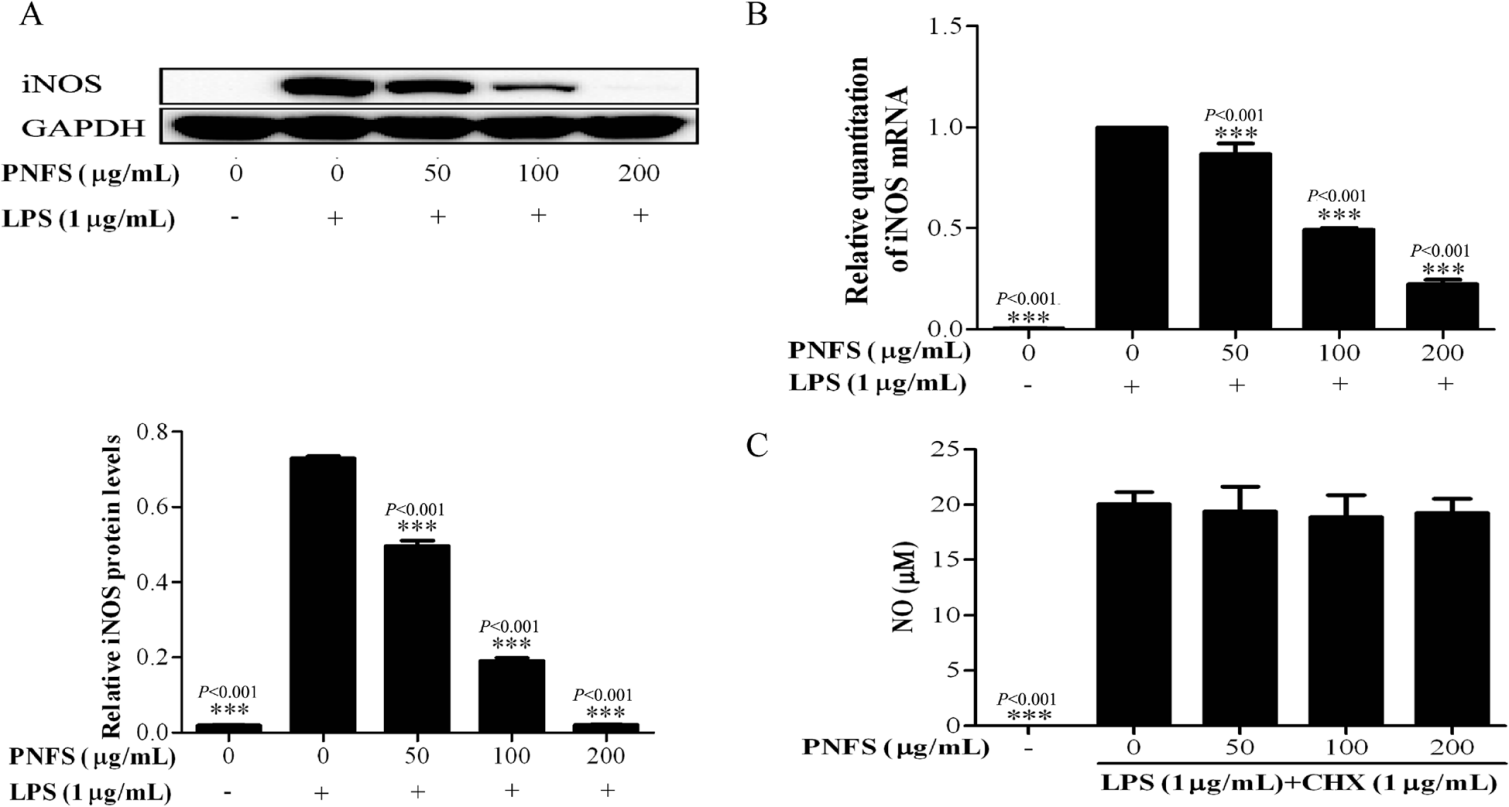
PNFS significantly inhibited the iNOS gene overexpression, but did not affect iNOS enzymatic activity in LPS-stimulated RAW264.7 macrophages. **a** PNFS inhibited the LPS-stimulated iNOS protein overproduction in RAW264.7 macrophages. **b** PNFS suppressed the LPS-stimulated iNOS mRNA overexpression in RAW264.7 macrophages. **c** PNFS did not affect LPS-stimulated iNOS enzymatic activity in Raw264.7 macrophages. Data in Fig. 4**a**, **b** and **c** were all expressed as the mean (SD) of 3 independent experiments. One-Way ANOVA test was used to analyzed the data and the results were *F = 1611.288; P < 0.001*, *F = 414.434; P < 0.001* and *F = 110.064; P < 0.001*, respectively. Then, data in Fig. 4**a**, **b** and **c** were all counted by SNK and LSD multiple comparisons to determine the statistical difference between two groups. The *P* values represented the statistical differences between each group and the corresponding positive control (without PNFS and with LPS treated). *** means *P < 0.001*

We next assessed whether PNFS inhibited NO overproduction by suppressing iNOS gene overexpression using western blotting and real-time PCR to measure total proteins and total RNA, respectively. iNOS protein and mRNA levels were significantly increased after stimulated by LPS, while PNFS (50, 100, 200 μg/mL) apparently inhibited iNOS protein overproduction (*P <* 0.001, *P <* 0.001, *P <* 0.001) and iNOS mRNA overexpression (*P <* 0.001, *P <* 0.001, *P <* 0.001) (Fig. 4a and b). This suggested that PNFS could decrease NO overproduction *via* inhibiting iNOS gene overexpression.

We then clarified whether PNFS-mediated suppression of NO overproduction was also caused by lowering iNOS catalytic activity. LPS (1 μg/mL) was used to activate RAW264.7 macrophages for 24 h first, then cells were washed and treated with CHX (1 μg/ml) and PNFS (0, 50, 100, 200 μg/mL) for the next 24 h. iNOS proteins had already been induced in 24 h-LPS-stimulated cells, but their further production was blocked by adding CHX, which is a translation inhibitor. Therefore, NO output only depended on the iNOS catalytic activity. PNFS (50, 100, 200 μg/mL) did not change the nitrite levels in cells (Fig. 4c). This suggested that PNFS did not affect the iNOS enzymatic activity in LPS-stimulated RAW264.7 macrophages.

### PNFS significantly inhibits the overexpression of LPS-stimulated TLR4 mRNA in RAW264.7 macrophages

**Fig. 5:**
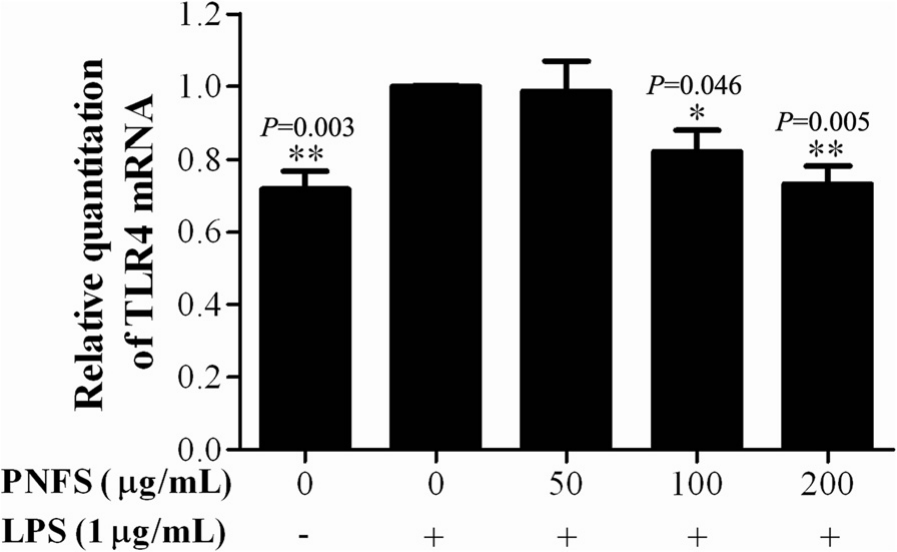
PNFS significantly inhibited the overexpression of LPS-stimulated TLR4 mRNA in RAW264.7 macrophages. Data were expressed as the mean (SD) of 3 independent experiments. One-Way ANOVA test was used to analyzed the data and the result was *F = 5.705; P = 0.003*. Then, data were counted by SNK and LSD multiple comparisons to determine the statistical difference between two groups. The *P* values represented the statistical differences between each group and the corresponding positive control (without PNFS and with LPS treated). * means *P < 0.05* and ** means *P < 0.01*

iNOS gene expression is mainly induced *via* activation of the TLR4 signaling pathway. Total RNA was separated for real-time PCR to detect the inhibitory effects of PNFS (0, 50, 100, 200 μg/mL) on LPS-induced TLR4 overexpression in macrophages. PNFS (100, 200 μg/mL) significantly inhibited LPS-induced TLR4 mRNA overexpression (*P =* 0.046, *P =* 0.005) (Fig. 5).

### PNFS markedly suppresses activation of LPS-stimulated MAPK signaling pathway in RAW264.7 macrophages

**Fig. 6:**
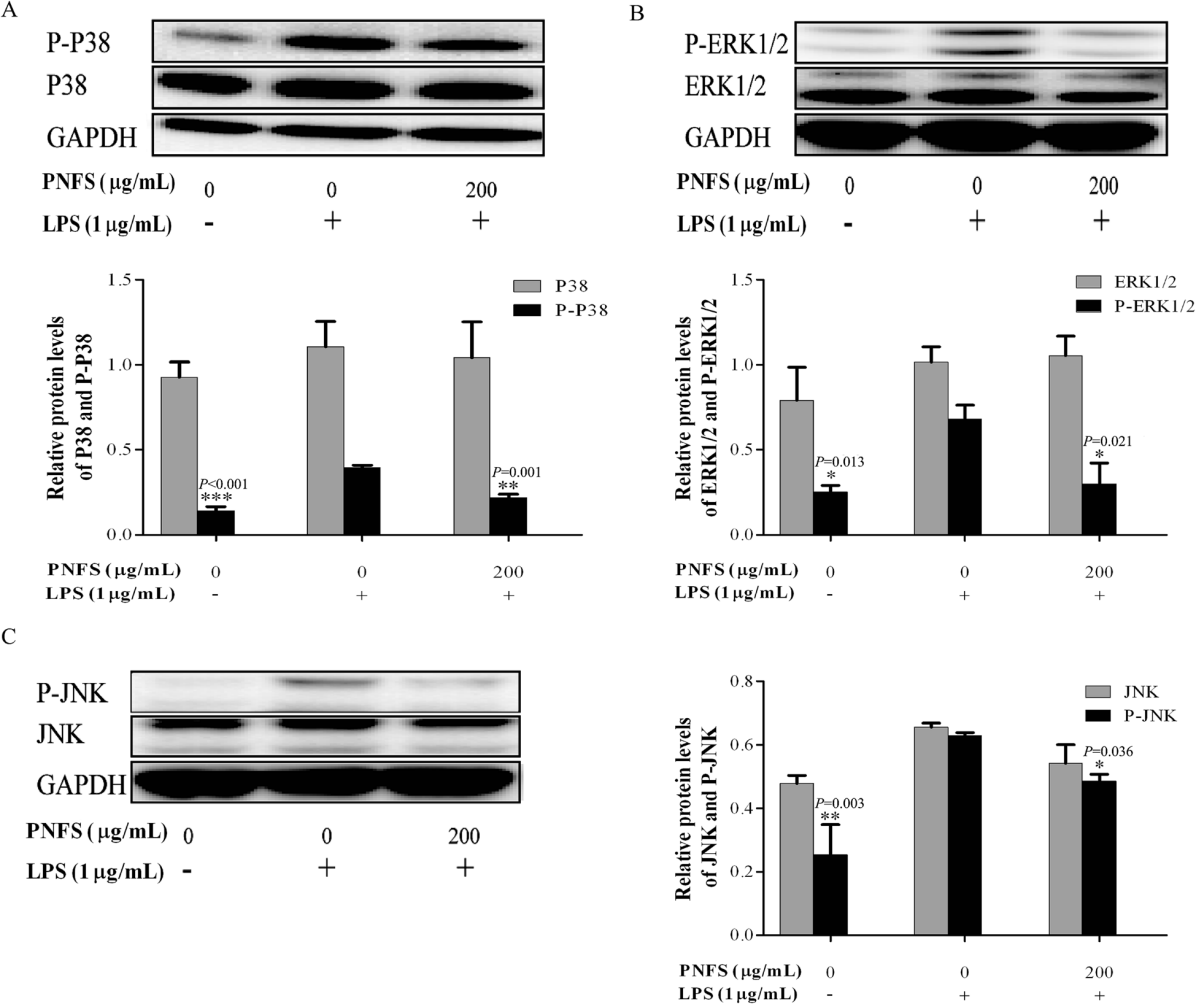
PNFS markedly suppressed the activation of LPS-stimulated MAPK signaling pathway in RAW264.7 macrophages. **a** PNFS suppressed the P38 activation in LPS-stimulated RAW264.7 macrophages. **b** PNFS suppressed the ERK1/2 activation in LPS-stimulated RAW264.7 macrophages. **c** PNFS suppressed the JNK activation in LPS-stimulated RAW264.7 macrophages. Data of the production of P-P38, total P38, P-ERK1/2, total ERK1/2, P-JNK and total JNK were all expressed as the mean (SD) of 3 independent experiments. One-Way ANOVA test was used to analyzed the data and the results were *F = 45.104; P < 0.001*, *F = 0.322; P = 0.736*, *F = 7.330; P = 0.024*, *F = 1.019; P = 0.416*, *F = 11.180; P = 0.009* and *F = 2.144; P = 0.198*, respectively. Then, data of the production of P-P38, total P38, P-ERK1/2, total ERK1/2, P-JNK and total JNK were all counted by SNK and LSD multiple comparisons to determine the statistical difference between two groups. The *P* values represented the statistical differences between each group and the corresponding positive control (without PNFS and with LPS treated). * means *P < 0.05*, ** means *P < 0.01* and *** means *P < 0.001*

Based on the results described above, PNFS (200 μg/mL) had no cytotoxic effect on RAW264.7 macrophages and showed the best anti-inflammatory efficacy. RAW264.7 macrophages were treated with LPS (1 μg/ml) and PNFS (0, 200 μg/mL) for 3 h. Then, western blotting was used to detect the levels of phospho-P38 (P-P38), phospho-ERK1/2 (P-ERK1/2) and phospho-JNK (P-JNK), along with the corresponding total P38, ERK1/2 and JNK. LPS obviously increased MAPKs activation by increasing the phosphorylation of P38, ERK1/2 and JNK in LPS-stimulated RAW264.7 macrophages. The overproduction of P-P38, P-ERK1/2 and P-JNK was markedly inhibited by PNFS (200 μg/mL) and the average inhibition rates were 45 %, 56 % and 23 % (*P =* 0.001, *P =* 0.021, *P = 0.*036), respectively (Fig. 6a, b and c). By contrast, PNFS (200 μg/mL) did not significantly affect the production of total P38, ERK1/2 and JNK. These results demonstrated that PNFS was able to reduce iNOS gene overexpression by inhibiting the activation of MAPK signaling pathway.

### PNFS does not activate the LPS-stimulated PI3K/Akt signaling pathway in RAW264.7 macrophages

**Fig. 7:**
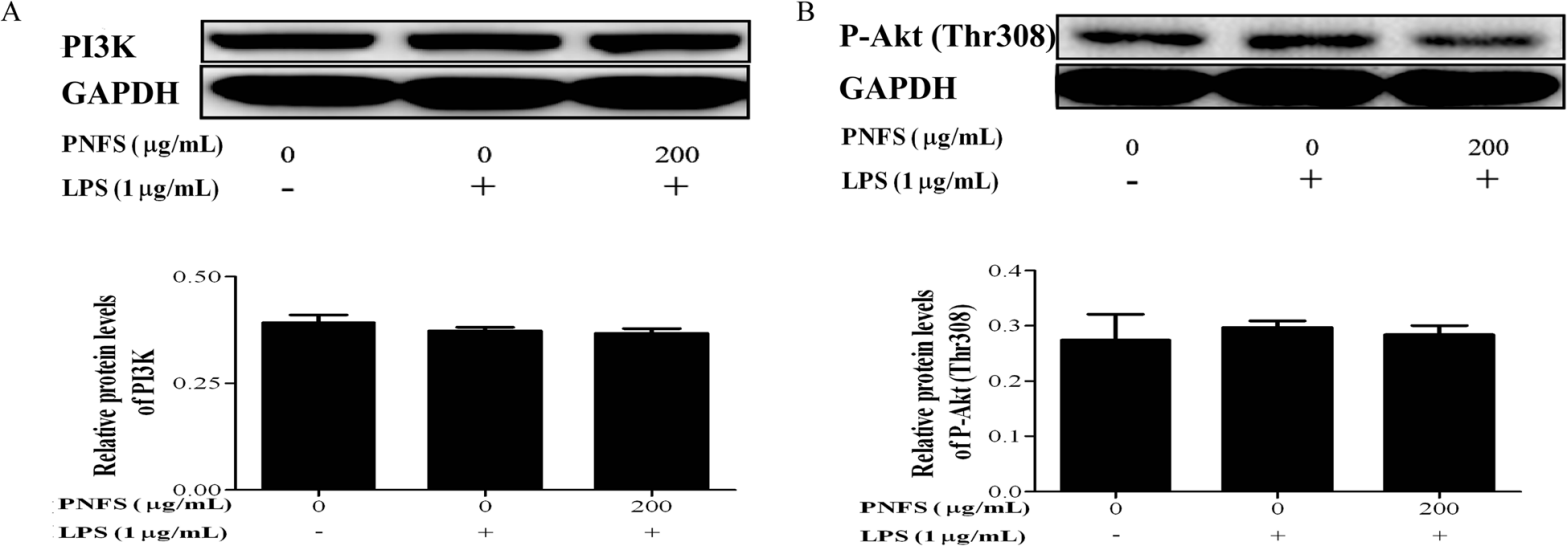
PNFS did not activate LPS-stimulated PI3K/Akt signaling pathway in RAW264.7 macrophages. **a** PNFS had no effect on PI3K production in LPS-stimulated RAW264.7 macrophages. **b** PNFS had no effect on P-Akt (Thr308) production in LPS-stimulated RAW264.7 macrophages. Data in Fig. 7**a** and **b** were both expressed as the mean (SD) of 3 independent experiments. One-Way ANOVA test was used to analyzed the data and the results were *F = 0.999; P = 0.422* and *F = 0.157; P = 0.858*, respectively. Then, data in Fig. 7**a** and **b** were both counted by SNK and LSD multiple comparisons to determine the statistical difference between two groups. The *P* values represented the statistical differences between each group and the corresponding positive control (without PNFS and with LPS treated)

iNOS gene expression can be suppressed *via* activating the PI3K/Akt signaling pathway [27]. In this study, the effects of PNFS on the LPS-stimulated PI3K/Akt signaling pathway in RAW264.7 macrophages were evaluated. After cells were incubated with LPS (1 μg/mL) and PNFS (0, 200 μg/mL) for 3 h, we used western blotting to measure the levels of PI3K and phospho-Akt (P-Akt) (Thr308). PNFS (200 μg/mL) did not affect levels of PI3K or P-Akt (Thr308) (Fig. 7a and b), suggesting that the PI3K/Akt signaling pathway might not be involved in the mechanism by which PNFS suppresses iNOS gene overexpression.

### PNFS significantly inhibits activation of the LPS-stimulated NF-kappa B signaling pathway in RAW264.7 macrophages

**Fig. 8:**
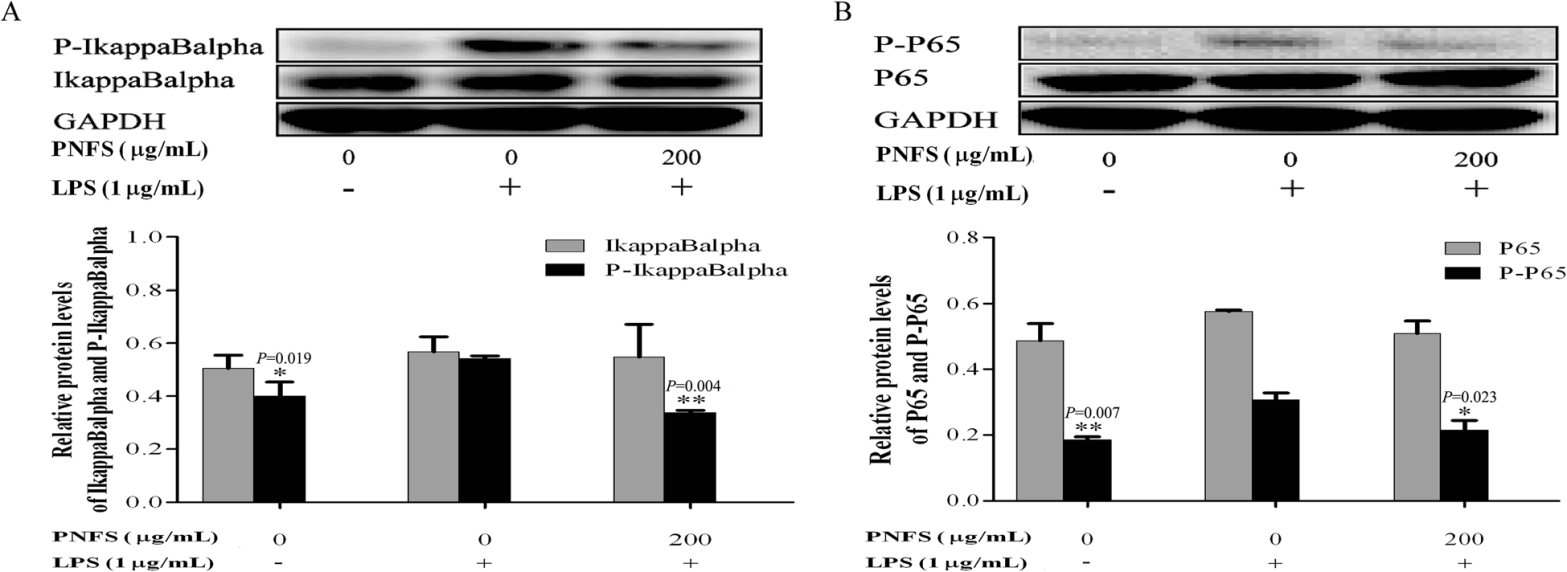
PNFS significantly inhibited the activation of LPS-stimulated NF-kappaB signaling pathway in RAW264.7 macrophages. **a** PNFS inhibited the IkappaBalpha activation in LPS-stimulated RAW264.7 macrophages. **b** PNFS inhibited the P65 activation in LPS-stimulated RAW264.7 macrophages. Data of the production of P-IkappaBalpha, total IkappaBalpha, P-P65 and total P65 were all expressed as the mean (SD) of 3 independent experiments. One-Way ANOVA test was used to analyzed the data and the results were *F = 11.172; P = 0.009*, *F = 0.146; P = 0.867*, *F = 8.793; P = 0.016* and *F = 0.062; P = 0.941*, respectively. Then, data of the production of P-IkappaBalpha, total IkappaBalpha, P-P65 and total P65 were all counted by SNK and LSD multiple comparisons to determine the statistical difference between two groups. The *P* values represented the statistical differences between each group and the corresponding positive control (without PNFS and with LPS treated). * means *P < 0.05* and ** means *P < 0.01*

To further explain the mechanisms underlying the inhibition of iNOS gene overexpression by PNFS, we studied the effects of PNFS on activation of the NF-kappa B signaling pathway. After cells were treated with PNFS (0, 200 μg/mL) and LPS (1 μg/mL) for 3 h, we used western blotting to examine the phosphorylation of I-kappa B alpha and P65 and the production of corresponding total I-kappa B alpha and P65. The production of P-I-kappa B alpha and P-P65 were significantly enhanced after cells were challenged with LPS for 3 h (*P =* 0.019, *P =* 0.007). Treatment with PNFS (200 μg/mL) apparently inhibited the overproduction of P-I-kappa B alpha and P-P65 and the average inhibition rates were 38 % and 30 % (*P =* 0.004, *P =* 0.023), respectively (Fig. 8a and b). However, PNFS (200 μg/mL) had no obvious effect on the production of total I-kappa B alpha and P65. These results suggested that PNFS could reduce iNOS gene overexpression by inhibiting NF-kappa B activation.

## Discussion

Our study demonstrates, for the first time, the suppressive effects of PNFS on LPS-stimulated NO overproduction and iNOS gene overexpression in RAW264.7 macrophages. These results also partially show the mechanisms underlying the anti-inflammatory effects of PNF, and provide a theoretical basis for the clinical treatment of inflammatory diseases with PNF.

When macrophages are stimulated with LPS, they become activated and then release various proinflammatory factors and cytokines such as IL-6, TNF-alpha and NO, whose excessive production results in extensive tissue damage and pathological changes. PNFS could significantly reduce NO and IL-6 overproduction, but did not exert any influence on TNF-alpha gene expression. As the inhibitory effect of PNFS on NO was more apparent than on IL-6, we focused on NO in this study.

NO is produced *via* decomposition of L-arginine in a reaction catalyzed by NOSs. eNOS and nNOS are constitutively expressed, while iNOS is mainly synthesized by activated macrophages [28]. When stimulated with LPS, iNOS gene expression is increased and macrophages release excessive NO. PNFS could evidently reduce LPS-stimulated NO overproduction via potent inhibition of iNOS gene overexpression, instead of suppressing iNOS enzymatic activity.

TLR4 triggers activation of MAPK/NF-kappa B signaling pathways to induce iNOS gene overexpression [29], and the TLR4-mediated signaling pathway also rapidly activates the PI3K/Akt signaling pathway to negatively regulate iNOS gene expression [23, 30]. PNFS had an obvious suppressive effect on LPS-activated TLR4 mRNA overexpression, suggesting that PNFS could inhibit the overproduction of NO and the overexpression of iNOS by blocking the TLR4 signaling pathway.

MAPKs transduce signals from the cell surface to the nucleus after being activated by various extracellular stimuli [31]. There are three parallel MAPK signaling pathways in mammalian cells, the P38 signaling pathway, the extracellular signal-regulated kinase (ERK) signaling pathway and the c-Jun N-terminal kinase (JNK) signaling pathway [32]. NF-kappa B, a common transcription factor, regulates various genes encoding inflammatory mediators and acts an important downstream target of MAPK signaling pathways in inflammatory and immune responses [33, 34]. NF-kappa B-activating stimuli, such as LPS and proinflammatory factors, induce I-kappa B phosphorylation, leading to their rapid degradation through the ubiquitin–proteasome pathway. As a result, NF-kappa B is translocated to the nucleus, and phosphorylated P65 (P-P65), which is a subunit of NF-kappa B, triggers iNOS gene expression [35]. PNFS significantly suppressed the I-kappa B alpha phosphorylation and degradation induced by LPS, thereby inhibiting NF-kappa B activation and reducing P65 phosphorylation. Additionally, PNFS attenuated LPS-induced phosphorylation of all three MAPKs studied. These findings suggest that PNFS inactivated P38, ERK1/2 and JNK, and then suppressed the NF-kappa B signaling pathway to prohibit iNOS gene overexpression.

PI3K catalyzes the generation of phosphatidylinositol 3, 4, 5-triphosphate (PIP3), and then PIP3 induces phosphorylation of Akt [36]. The generation of phospho-Akt (P-Akt) indicates the activation of the PI3K/Akt signaling pathway. PI3K is a negative regulator of iNOS gene expression [27]. The suppression of PI3K gene expression augments LPS-stimulated iNOS production in macrophages [23]. PNFS did not affect LPS-activated PI3K generation or P-Akt (Thr308) levels, indicating that PNFS did not suppress iNOS gene overexpression *via* activation of the PI3K/Akt signaling pathway.

## Conclusions

PNFS significantly down-regulated iNOS gene overexpression, and thereby decreased NO overproduction via the inhibition of TLR4-mediated MAPK/NF-kappa B signaling pathways, but not the PI3K/Akt signaling pathway.

## Abbreviations

CCK-8: Cell Counting Kit-8
CHX: Cycloheximide
DAF-FM DA: 3-Amino, 4-aminomethyl-2′,7′-difluorescein, diacetate
DMEM: Dulbecco’s modified Eagle medium
ECL: Chemiluminescence
ELISA: Enzyme-linked immunosorbent assay
eNOS: Endothelial nitric oxide synthase
ERK1/2: Extracellular-signal regulated kinase 1/2
FBS: Fetal bovine serum
I-kappa B: Inhibitor of NF-kappa B
IL-6: Interleukin-6
iNOS: Inducible nitric oxide synthase
JNK: c-Jun N-terminal kinase
LSD: Least significant difference
LPS: Lipopolysaccharide
MAPKs: Mitogen-activated protein kinases
NF-kappa B: Nuclear factor-kappa B
nNOS: neuronal nitric oxide synthase
NO: Nitric oxide
NO_2_^−^: Nitrite
NOSs: Nitric oxide synthases
PI3K: Phosphatidylinositol 3-kinase
PIP3: Phosphatidylinositol 3, 4, 5-triphosphate
PN: *Panax notoginseng* (Burk) F. H. Chen
PNF: *Panax notoginseng* (Burk) F. H. Chen flower bud
PNFS: *Panax Notoginseng* flower saponins
PNS: *Panax notoginseng* saponins
Real-time RT-PCR: Real-time quantitative reverse transcription-polymerase chain reaction
SNK: Student-Newman-Keuls
TLR4: Toll-like receptor 4
TNF-alpha: Tumor necrosis factor-alpha
